# *catena*-Poly[[tribenzyl­tin(IV)]-μ-4-nitro­cinnamato-κ^2^ *O*:*O*′]

**DOI:** 10.1107/S1600536808032236

**Published:** 2008-10-11

**Authors:** Pui Yee Thong, Kong Mun Lo, Seik Weng Ng

**Affiliations:** a Department of Chemistry, University of Malaya, 50603 Kuala Lumpur, Malaysia

## Abstract

The 4-nitro­cinnamate anion in the title compound, [Sn(C_7_H_7_)_3_(C_9_H_6_NO_4_)]_*n*_, bridges adjacent tribenzyl­tin cations into a helical chain running along the *b* axis. The Sn atoms in the two independent mol­ecules adopt distorted *trans*-C_3_SnO_2_ trigonal–bipyramidal geometries. The repeat distance of the polymeric chain is *b*/2.

## Related literature

The trialkyltin derivatives of monocarboxylic acids generally adopt carboxyl­ate-bridged chain structures; see: Ng *et al.* (1989 ▶). For the trialkyl­tin/triaryl­tin derivatives of cinnamic acid and other substituted cinnamic acids, see: Ng & Kumar Das (1994 ▶); Siah *et al.* (1994 ▶); Willem *et al.* (1996 ▶). For the synthesis of tribenzyl­tin chloride, see: Sisido *et al.* (1961 ▶). For reviews of organotin carboxyl­ates, see: Tiekink (1991 ▶, 1994 ▶).
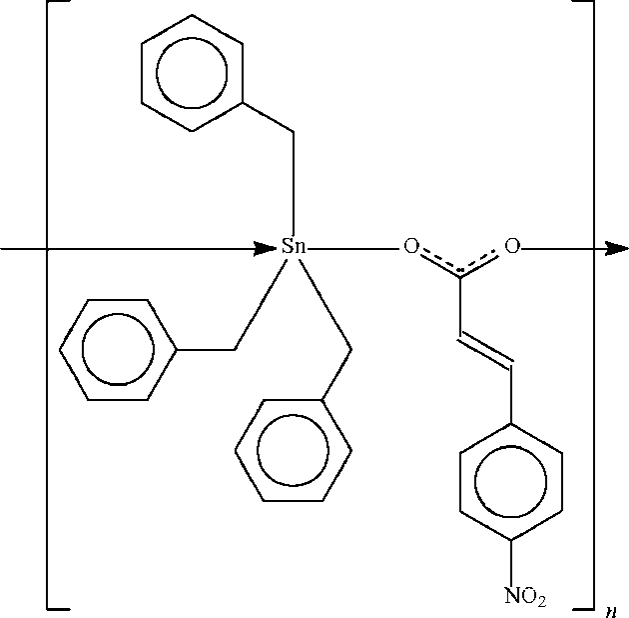


## Experimental

### 

#### Crystal data

[Sn(C_7_H_7_)_3_(C_9_H_6_NO_4_)]
*M* *_r_* = 584.22
Monoclinic, 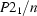

*a* = 21.4850 (3) Å
*b* = 10.2134 (1) Å
*c* = 25.9562 (4) Å
β = 112.133 (1)°
*V* = 5276.0 (1) Å^3^
*Z* = 8
Mo *K*α radiation
μ = 1.00 mm^−1^
*T* = 100 (2) K
0.36 × 0.03 × 0.03 mm

#### Data collection

Bruker SMART APEX diffractometer
Absorption correction: multi-scan (*SADABS*; Sheldrick, 1996 ▶) *T*_min_ = 0.714, *T*_max_ = 0.971
48779 measured reflections
12115 independent reflections
9043 reflections with *I* > 2σ(*I*)
*R*_int_ = 0.032

#### Refinement

*R*[*F* ^2^ > 2σ(*F* ^2^)] = 0.033
*wR*(*F* ^2^) = 0.091
*S* = 1.06
12115 reflections
565 parameters
38 restraints
H-atom parameters constrained
Δρ_max_ = 0.81 e Å^−3^
Δρ_min_ = −0.80 e Å^−3^

### 

Data collection: *APEX2* (Bruker, 2007 ▶); cell refinement: *SAINT* (Bruker, 2007 ▶); data reduction: *SAINT*; program(s) used to solve structure: *SHELXS97* (Sheldrick, 2008 ▶); program(s) used to refine structure: *SHELXL97* (Sheldrick, 2008 ▶); molecular graphics: *X-SEED* (Barbour, 2001 ▶); software used to prepare material for publication: *publCIF* (Westrip, 2008 ▶).

## Supplementary Material

Crystal structure: contains datablocks global, I. DOI: 10.1107/S1600536808032236/tk2314sup1.cif

Structure factors: contains datablocks I. DOI: 10.1107/S1600536808032236/tk2314Isup2.hkl

Additional supplementary materials:  crystallographic information; 3D view; checkCIF report

## Figures and Tables

**Fig. 1.**
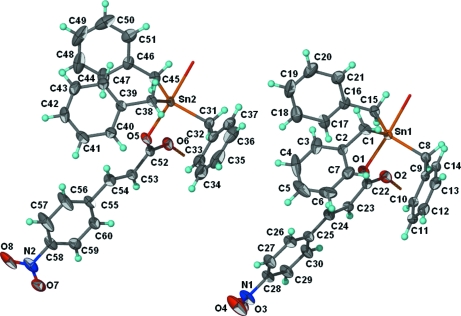
70% Probability thermal ellipsoid plot (Barbour, 2001) of [(C6H5CH2)3SnO2CCH═CH—C6H4—NO2]∞. Hydrogen atoms are drawn as spheres of arbitrary radius. Only one orientation of the disordered nitro group is shown in each case.

## supplementary crystallographic information

### Comment

The polymeric structure found for tribenzyl(4-nitrocinnamato)tin(IV) conforms to expectation, Fig. 1 (Ng *et al.*, 1989; Tiekink, 1991).

### Experimental

Tribenzyltin chloride was synthesized from the direct reaction between tin powder and benzyl chloride in water (Sisido *et al.*, 1961). The chloride was converted to tribenzyltin hydroxide by treatment with 10% sodium hydroxide in acetone. Tribenzyltin hydroxide (1.4 g, 0.3 mmol) and 4-nitrocinnamic acid (0.5 g, 0.3 mmol) were heated in ethanol (100 ml) for 1 h. Slow evaporation of the solvent yielded yellow prisms.

### Refinement

The carbon-bound H-atoms were placed in calculated positions (C—H 0.95–0.99 Å) and were included in the refinement in the riding model approximation, with *U*(H) set to 1.2*U*_eq_(C). The nitro group in each independent molecule are disordered in their oxygen atoms. The nitrogen–oxygen distances for the unprimed and primed atoms were restrained to within 0.01 Å of each other. The C–NO_2_ fragment was restrained to be nearly planar, and the temperature factors of the primed atoms were set to those of the unprimed ones; the anisotropic displacement parameters of the disordered atoms were restrained to be nearly isotropic. The occupancy could not be refined, and was arbitrarily set at 0.5. All phenyl and phenylene rings were refined as rigid hexagons of 1.39 Å sides.

### Crystal data

**Table:**

[Sn(C_7_H_7_)_3_(C_9_H_6_NO_4_)]	*F*(000) = 2368
*M**_r_* = 584.22	*D*_x_ = 1.471 Mg m^−^^3^
Monoclinic, *P*2_1_/*n*	Mo *K*α radiation, λ = 0.71073 Å
Hall symbol: -P 2yn	Cell parameters from 9977 reflections
*a* = 21.4850 (3) Å	θ = 2.4–28.3°
*b* = 10.2134 (1) Å	µ = 1.00 mm^−^^1^
*c* = 25.9562 (4) Å	*T* = 100 K
β = 112.133 (1)°	Prism, pale yellow
*V* = 5276.0 (1) Å^3^	0.36 × 0.03 × 0.03 mm
*Z* = 8	

### Data collection

**Table:**

Bruker SMART APEX diffractometer	12115 independent reflections
Radiation source: fine-focus sealed tube	9043 reflections with *I* > 2σ(*I*)
graphite	*R*_int_ = 0.032
ω scans	θ_max_ = 27.5°, θ_min_ = 1.1°
Absorption correction: multi-scan (SADABS; Sheldrick, 1996)	*h* = −26→27
*T*_min_ = 0.714, *T*_max_ = 0.971	*k* = −13→13
48779 measured reflections	*l* = −32→33

### Refinement

**Table:**

Refinement on *F*^2^	Primary atom site location: structure-invariant direct methods
Least-squares matrix: full	Secondary atom site location: difference Fourier map
*R*[*F*^2^ > 2σ(*F*^2^)] = 0.033	Hydrogen site location: inferred from neighbouring sites
*wR*(*F*^2^) = 0.091	H-atom parameters constrained
*S* = 1.06	*w* = 1/[σ^2^(*F*_o_^2^) + (0.0362*P*)^2^ + 8.7819*P*] where *P* = (*F*_o_^2^ + 2*F*_c_^2^)/3
12115 reflections	(Δ/σ)_max_ = 0.001
565 parameters	Δρ_max_ = 0.81 e Å^−^^3^
38 restraints	Δρ_min_ = −0.80 e Å^−^^3^

### Fractional atomic coordinates and isotropic or equivalent isotropic displacement parameters (Å^2^)

**Table:**

	*x*	*y*	*z*	*U*_iso_*/*U*_eq_	Occ. (<1)
Sn1	0.740576 (10)	0.624465 (18)	0.773616 (8)	0.01626 (6)	
Sn2	0.240598 (10)	0.527516 (18)	0.774258 (8)	0.01660 (6)	
O1	0.72572 (11)	0.46521 (19)	0.82387 (9)	0.0208 (4)	
O2	0.74811 (12)	0.3133 (2)	0.77179 (9)	0.0285 (5)	
O3	0.5986 (4)	−0.1699 (6)	1.0109 (5)	0.0358 (16)	0.50
O4	0.6223 (5)	−0.0096 (19)	1.0718 (4)	0.063 (2)	0.50
O3'	0.6225 (4)	−0.1780 (6)	1.0239 (5)	0.0358 (16)	0.50
O4'	0.5957 (5)	−0.0086 (19)	1.0602 (4)	0.063 (2)	0.50
O5	0.22693 (12)	0.36739 (19)	0.82563 (9)	0.0239 (5)	
O6	0.24714 (11)	0.2147 (2)	0.77260 (9)	0.0273 (5)	
O7	0.1482 (10)	−0.2711 (10)	1.0365 (13)	0.039 (2)	0.50
O8	0.0768 (8)	−0.119 (3)	1.0434 (7)	0.047 (3)	0.50
O7'	0.1347 (10)	−0.2814 (10)	1.0294 (13)	0.039 (2)	0.50
O8'	0.0890 (8)	−0.125 (3)	1.0558 (7)	0.047 (3)	0.50
N1	0.61765 (12)	−0.0592 (3)	1.02706 (11)	0.0367 (7)	
N2	0.11891 (13)	−0.1650 (3)	1.02647 (10)	0.0353 (7)	
C1	0.71495 (17)	0.7671 (3)	0.82323 (13)	0.0229 (6)	
H1A	0.7535	0.8275	0.8394	0.027*	
H1B	0.6765	0.8193	0.7984	0.027*	
C2	0.69693 (10)	0.7136 (2)	0.86959 (7)	0.0190 (6)	
C3	0.63172 (9)	0.6732 (2)	0.85994 (8)	0.0312 (8)	
H3	0.5977	0.6815	0.8239	0.037*	
C4	0.61630 (11)	0.6204 (2)	0.90307 (12)	0.0484 (12)	
H4	0.5717	0.5928	0.8965	0.058*	
C5	0.66609 (15)	0.6082 (2)	0.95584 (10)	0.0544 (14)	
H5	0.6555	0.5721	0.9853	0.065*	
C6	0.73130 (13)	0.6487 (3)	0.96549 (7)	0.0502 (12)	
H6	0.7653	0.6403	1.0016	0.060*	
C7	0.74672 (8)	0.7014 (2)	0.92237 (8)	0.0320 (8)	
H7	0.7913	0.7290	0.9290	0.038*	
C8	0.84579 (15)	0.5814 (3)	0.79622 (13)	0.0225 (6)	
H8A	0.8517	0.4871	0.7904	0.027*	
H8B	0.8637	0.6322	0.7724	0.027*	
C9	0.88404 (10)	0.61648 (18)	0.85682 (6)	0.0203 (6)	
C10	0.88920 (10)	0.52659 (14)	0.89844 (8)	0.0227 (6)	
H10	0.8693	0.4424	0.8890	0.027*	
C11	0.92346 (10)	0.55980 (18)	0.95392 (7)	0.0257 (7)	
H11	0.9270	0.4984	0.9824	0.031*	
C12	0.95257 (10)	0.6829 (2)	0.96778 (6)	0.0278 (7)	
H12	0.9760	0.7056	1.0057	0.033*	
C13	0.94741 (10)	0.77282 (15)	0.92616 (8)	0.0266 (7)	
H13	0.9673	0.8570	0.9356	0.032*	
C14	0.91314 (10)	0.73960 (16)	0.87068 (7)	0.0219 (6)	
H14	0.9096	0.8010	0.8422	0.026*	
C15	0.66090 (15)	0.5625 (3)	0.69825 (13)	0.0215 (6)	
H15A	0.6636	0.6116	0.6663	0.026*	
H15B	0.6660	0.4681	0.6920	0.026*	
C16	0.59325 (9)	0.5861 (2)	0.70202 (9)	0.0234 (7)	
C17	0.56721 (10)	0.49508 (17)	0.72834 (9)	0.0270 (7)	
H17	0.5912	0.4166	0.7428	0.032*	
C18	0.50613 (11)	0.5189 (2)	0.73352 (10)	0.0362 (9)	
H18	0.4883	0.4567	0.7515	0.043*	
C19	0.47109 (9)	0.6337 (3)	0.71236 (11)	0.0442 (10)	
H19	0.4293	0.6500	0.7159	0.053*	
C20	0.49713 (11)	0.7247 (2)	0.68604 (11)	0.0467 (10)	
H20	0.4732	0.8032	0.6716	0.056*	
C21	0.55821 (11)	0.70092 (19)	0.68086 (10)	0.0352 (8)	
H21	0.5760	0.7631	0.6629	0.042*	
C22	0.73041 (15)	0.3466 (3)	0.81149 (12)	0.0193 (6)	
C23	0.71423 (15)	0.2458 (3)	0.84523 (12)	0.0200 (6)	
H23	0.7156	0.1561	0.8361	0.024*	
C24	0.69782 (15)	0.2774 (3)	0.88809 (13)	0.0222 (6)	
H24	0.6986	0.3679	0.8967	0.027*	
C25	0.67857 (11)	0.18658 (16)	0.92365 (8)	0.0217 (6)	
C26	0.66231 (12)	0.24047 (13)	0.96615 (9)	0.0273 (7)	
H26	0.6651	0.3324	0.9721	0.033*	
C27	0.64194 (12)	0.15980 (18)	1.00000 (8)	0.0300 (8)	
H27	0.6308	0.1966	1.0290	0.036*	
C28	0.63784 (12)	0.02523 (17)	0.99136 (8)	0.0260 (7)	
C29	0.65410 (13)	−0.02866 (13)	0.94886 (9)	0.0290 (8)	
H29	0.6513	−0.1206	0.9430	0.035*	
C30	0.67447 (12)	0.05201 (17)	0.91501 (8)	0.0279 (7)	
H30	0.6856	0.0152	0.8860	0.034*	
C31	0.34371 (15)	0.4752 (3)	0.78971 (13)	0.0234 (7)	
H31A	0.3489	0.3789	0.7916	0.028*	
H31B	0.3567	0.5082	0.7593	0.028*	
C32	0.38785 (10)	0.5362 (2)	0.84441 (7)	0.0257 (7)	
C33	0.39227 (11)	0.4797 (2)	0.89438 (9)	0.0348 (8)	
H33	0.3704	0.3988	0.8943	0.042*	
C34	0.42867 (12)	0.5415 (3)	0.94443 (7)	0.0456 (11)	
H34	0.4317	0.5029	0.9786	0.055*	
C35	0.46065 (12)	0.6599 (3)	0.94449 (8)	0.0482 (11)	
H35	0.4855	0.7021	0.9787	0.058*	
C36	0.45623 (11)	0.71638 (19)	0.89452 (11)	0.0425 (10)	
H36	0.4781	0.7973	0.8946	0.051*	
C37	0.41983 (11)	0.6546 (2)	0.84447 (8)	0.0327 (8)	
H37	0.4168	0.6932	0.8103	0.039*	
C38	0.23174 (16)	0.6679 (3)	0.83283 (13)	0.0207 (6)	
H38A	0.2751	0.6712	0.8650	0.025*	
H38B	0.2242	0.7552	0.8150	0.025*	
C39	0.17661 (9)	0.6429 (2)	0.85455 (8)	0.0206 (6)	
C40	0.18741 (8)	0.5606 (2)	0.89975 (8)	0.0258 (7)	
H40	0.2299	0.5198	0.9176	0.031*	
C41	0.13605 (11)	0.5380 (2)	0.91892 (8)	0.0325 (8)	
H41	0.1434	0.4817	0.9498	0.039*	
C42	0.07389 (9)	0.5977 (2)	0.89288 (9)	0.0349 (8)	
H42	0.0388	0.5822	0.9060	0.042*	
C43	0.06308 (8)	0.6800 (2)	0.84768 (9)	0.0376 (9)	
H43	0.0206	0.7208	0.8299	0.045*	
C44	0.11444 (10)	0.7026 (2)	0.82851 (8)	0.0293 (7)	
H44	0.1071	0.7589	0.7976	0.035*	
C45	0.15670 (16)	0.4719 (3)	0.70112 (14)	0.0245 (7)	
H45A	0.1653	0.4998	0.6679	0.029*	
H45B	0.1527	0.3753	0.7000	0.029*	
C46	0.09086 (9)	0.5305 (2)	0.69842 (10)	0.0258 (7)	
C47	0.05407 (12)	0.4697 (2)	0.72566 (10)	0.0370 (9)	
H47	0.0697	0.3901	0.7452	0.044*	
C48	−0.00562 (12)	0.5252 (3)	0.72425 (12)	0.0517 (12)	
H48	−0.0308	0.4836	0.7429	0.062*	
C49	−0.02853 (10)	0.6416 (3)	0.69561 (12)	0.0561 (13)	
H49	−0.0693	0.6795	0.6946	0.067*	
C50	0.00826 (13)	0.7024 (2)	0.66837 (11)	0.0506 (12)	
H50	−0.0074	0.7820	0.6488	0.061*	
C51	0.06795 (12)	0.6469 (2)	0.66978 (10)	0.0353 (8)	
H51	0.0931	0.6885	0.6512	0.042*	
C52	0.23218 (15)	0.2495 (3)	0.81304 (13)	0.0198 (6)	
C53	0.21856 (16)	0.1483 (3)	0.84854 (13)	0.0211 (6)	
H53	0.2298	0.0597	0.8450	0.025*	
C54	0.19105 (17)	0.1782 (3)	0.88532 (13)	0.0254 (7)	
H54	0.1828	0.2683	0.8893	0.030*	
C55	0.17221 (12)	0.08569 (17)	0.92023 (9)	0.0255 (7)	
C56	0.13401 (15)	0.13270 (16)	0.94915 (12)	0.0541 (13)	
H56	0.1202	0.2217	0.9454	0.065*	
C57	0.11599 (14)	0.0494 (2)	0.98357 (11)	0.0567 (14)	
H57	0.0899	0.0816	1.0033	0.068*	
C58	0.13617 (13)	−0.08082 (19)	0.98908 (9)	0.0283 (7)	
C59	0.17438 (12)	−0.12784 (15)	0.96017 (9)	0.0288 (7)	
H59	0.1882	−0.2169	0.9639	0.035*	
C60	0.19240 (11)	−0.04458 (19)	0.92574 (9)	0.0265 (7)	
H60	0.2185	−0.0767	0.9060	0.032*	

### Atomic displacement parameters (Å^2^)

**Table:**

	*U*^11^	*U*^22^	*U*^33^	*U*^12^	*U*^13^	*U*^23^
Sn1	0.02067 (11)	0.01290 (10)	0.01803 (10)	−0.00047 (8)	0.01050 (8)	−0.00080 (8)
Sn2	0.01892 (10)	0.01368 (10)	0.02153 (11)	0.00060 (8)	0.01256 (8)	−0.00001 (8)
O1	0.0309 (12)	0.0125 (10)	0.0231 (11)	−0.0006 (9)	0.0149 (10)	−0.0016 (8)
O2	0.0385 (14)	0.0240 (12)	0.0296 (12)	0.0031 (10)	0.0203 (11)	−0.0003 (10)
O3	0.046 (5)	0.0261 (15)	0.041 (4)	−0.009 (2)	0.023 (4)	0.0018 (18)
O4	0.120 (7)	0.0448 (19)	0.049 (4)	−0.019 (6)	0.060 (4)	−0.008 (3)
O3'	0.046 (5)	0.0261 (15)	0.041 (4)	−0.009 (2)	0.023 (4)	0.0018 (18)
O4'	0.120 (7)	0.0448 (19)	0.049 (4)	−0.019 (6)	0.060 (4)	−0.008 (3)
O5	0.0386 (13)	0.0129 (10)	0.0305 (12)	0.0013 (9)	0.0247 (11)	0.0013 (9)
O6	0.0357 (13)	0.0235 (12)	0.0315 (12)	0.0019 (10)	0.0228 (11)	−0.0018 (10)
O7	0.035 (7)	0.039 (2)	0.037 (6)	−0.014 (3)	0.008 (5)	0.013 (2)
O8	0.084 (5)	0.043 (3)	0.029 (6)	−0.018 (4)	0.040 (5)	−0.004 (6)
O7'	0.035 (7)	0.039 (2)	0.037 (6)	−0.014 (3)	0.008 (5)	0.013 (2)
O8'	0.084 (5)	0.043 (3)	0.029 (6)	−0.018 (4)	0.040 (5)	−0.004 (6)
N1	0.063 (2)	0.0263 (16)	0.0308 (16)	−0.0070 (15)	0.0295 (16)	−0.0007 (13)
N2	0.0500 (19)	0.0320 (17)	0.0264 (15)	−0.0184 (15)	0.0172 (14)	−0.0017 (13)
C1	0.0378 (18)	0.0084 (13)	0.0267 (16)	−0.0008 (12)	0.0169 (14)	−0.0032 (12)
C2	0.0235 (15)	0.0129 (13)	0.0246 (15)	0.0027 (11)	0.0135 (13)	−0.0024 (12)
C3	0.0278 (17)	0.0282 (17)	0.043 (2)	−0.0029 (14)	0.0192 (16)	−0.0153 (16)
C4	0.057 (3)	0.0265 (19)	0.091 (4)	−0.0157 (18)	0.062 (3)	−0.022 (2)
C5	0.108 (4)	0.0225 (19)	0.066 (3)	0.013 (2)	0.070 (3)	0.0097 (19)
C6	0.075 (3)	0.051 (3)	0.033 (2)	0.032 (2)	0.030 (2)	0.0167 (19)
C7	0.0285 (18)	0.042 (2)	0.0258 (17)	0.0099 (15)	0.0105 (14)	−0.0001 (15)
C8	0.0256 (16)	0.0215 (15)	0.0253 (16)	0.0038 (13)	0.0152 (13)	0.0028 (13)
C9	0.0186 (15)	0.0209 (15)	0.0253 (16)	0.0035 (12)	0.0127 (13)	0.0033 (12)
C10	0.0217 (15)	0.0198 (15)	0.0293 (17)	0.0035 (12)	0.0127 (13)	0.0056 (13)
C11	0.0248 (16)	0.0286 (17)	0.0275 (17)	0.0080 (14)	0.0140 (14)	0.0099 (14)
C12	0.0263 (17)	0.0344 (18)	0.0230 (16)	0.0053 (14)	0.0098 (14)	−0.0028 (14)
C13	0.0250 (16)	0.0219 (16)	0.0337 (18)	0.0021 (13)	0.0119 (14)	−0.0034 (14)
C14	0.0222 (15)	0.0189 (15)	0.0291 (16)	0.0037 (12)	0.0146 (13)	0.0057 (12)
C15	0.0237 (16)	0.0210 (15)	0.0223 (15)	−0.0019 (12)	0.0115 (13)	−0.0034 (12)
C16	0.0212 (16)	0.0246 (16)	0.0233 (16)	−0.0016 (13)	0.0071 (13)	−0.0064 (13)
C17	0.0287 (17)	0.0275 (17)	0.0257 (17)	−0.0075 (14)	0.0112 (14)	−0.0077 (14)
C18	0.0291 (19)	0.049 (2)	0.0323 (19)	−0.0132 (17)	0.0134 (16)	−0.0112 (17)
C19	0.0226 (18)	0.069 (3)	0.043 (2)	−0.0029 (18)	0.0144 (17)	−0.010 (2)
C20	0.031 (2)	0.054 (3)	0.054 (3)	0.0162 (19)	0.0138 (19)	0.002 (2)
C21	0.0315 (19)	0.034 (2)	0.040 (2)	0.0057 (15)	0.0131 (16)	0.0026 (16)
C22	0.0207 (15)	0.0162 (14)	0.0223 (15)	0.0007 (12)	0.0094 (12)	−0.0022 (12)
C23	0.0235 (15)	0.0118 (14)	0.0251 (15)	−0.0007 (11)	0.0097 (13)	0.0009 (11)
C24	0.0281 (16)	0.0139 (14)	0.0275 (16)	−0.0021 (12)	0.0137 (13)	−0.0006 (12)
C25	0.0275 (16)	0.0177 (14)	0.0230 (15)	−0.0006 (12)	0.0130 (13)	0.0005 (12)
C26	0.041 (2)	0.0175 (15)	0.0290 (17)	0.0022 (14)	0.0197 (15)	−0.0006 (13)
C27	0.046 (2)	0.0271 (17)	0.0273 (17)	0.0006 (15)	0.0255 (16)	−0.0036 (14)
C28	0.0375 (19)	0.0219 (16)	0.0228 (16)	−0.0024 (14)	0.0162 (15)	0.0008 (13)
C29	0.048 (2)	0.0177 (16)	0.0295 (18)	−0.0028 (14)	0.0237 (17)	−0.0017 (13)
C30	0.044 (2)	0.0207 (15)	0.0280 (17)	−0.0046 (14)	0.0242 (16)	−0.0037 (13)
C31	0.0234 (16)	0.0205 (15)	0.0306 (17)	0.0049 (12)	0.0152 (14)	0.0003 (13)
C32	0.0178 (15)	0.0264 (17)	0.0331 (18)	0.0069 (13)	0.0098 (14)	0.0017 (14)
C33	0.0247 (18)	0.048 (2)	0.0314 (19)	0.0064 (16)	0.0106 (15)	0.0059 (17)
C34	0.028 (2)	0.076 (3)	0.031 (2)	0.010 (2)	0.0098 (16)	0.003 (2)
C35	0.0255 (19)	0.068 (3)	0.043 (2)	0.008 (2)	0.0039 (17)	−0.020 (2)
C36	0.0274 (19)	0.035 (2)	0.058 (3)	0.0038 (16)	0.0083 (18)	−0.0115 (19)
C37	0.0241 (17)	0.0282 (18)	0.044 (2)	0.0077 (14)	0.0114 (16)	0.0036 (16)
C38	0.0283 (16)	0.0136 (14)	0.0254 (16)	0.0005 (12)	0.0160 (13)	−0.0013 (12)
C39	0.0265 (16)	0.0176 (15)	0.0214 (15)	0.0026 (12)	0.0133 (13)	−0.0015 (12)
C40	0.0308 (17)	0.0246 (16)	0.0266 (17)	0.0092 (14)	0.0161 (14)	0.0039 (13)
C41	0.050 (2)	0.0233 (17)	0.0348 (19)	0.0058 (15)	0.0287 (18)	0.0055 (14)
C42	0.034 (2)	0.042 (2)	0.041 (2)	−0.0001 (16)	0.0278 (17)	−0.0043 (17)
C43	0.0273 (18)	0.056 (2)	0.0338 (19)	0.0129 (17)	0.0167 (16)	0.0014 (18)
C44	0.0311 (18)	0.0361 (19)	0.0250 (17)	0.0106 (15)	0.0155 (14)	0.0043 (14)
C45	0.0260 (16)	0.0216 (16)	0.0281 (17)	−0.0034 (13)	0.0127 (14)	−0.0052 (13)
C46	0.0207 (16)	0.0269 (17)	0.0284 (17)	−0.0036 (13)	0.0076 (14)	−0.0088 (13)
C47	0.033 (2)	0.034 (2)	0.051 (2)	−0.0086 (16)	0.0228 (18)	−0.0098 (17)
C48	0.029 (2)	0.066 (3)	0.066 (3)	−0.012 (2)	0.024 (2)	−0.022 (2)
C49	0.024 (2)	0.079 (3)	0.056 (3)	0.012 (2)	0.0045 (19)	−0.026 (3)
C50	0.043 (2)	0.047 (3)	0.043 (2)	0.019 (2)	−0.0054 (19)	−0.011 (2)
C51	0.033 (2)	0.033 (2)	0.0335 (19)	0.0032 (16)	0.0055 (16)	−0.0051 (16)
C52	0.0217 (15)	0.0124 (14)	0.0301 (16)	−0.0002 (11)	0.0151 (13)	0.0011 (12)
C53	0.0278 (16)	0.0114 (13)	0.0265 (16)	−0.0008 (12)	0.0130 (13)	0.0007 (12)
C54	0.0351 (18)	0.0152 (14)	0.0302 (17)	−0.0009 (13)	0.0173 (15)	−0.0025 (13)
C55	0.0369 (19)	0.0210 (15)	0.0247 (16)	−0.0049 (14)	0.0185 (15)	−0.0006 (13)
C56	0.106 (4)	0.0200 (18)	0.075 (3)	0.008 (2)	0.078 (3)	0.0062 (19)
C57	0.110 (4)	0.027 (2)	0.073 (3)	0.003 (2)	0.080 (3)	0.002 (2)
C58	0.041 (2)	0.0245 (17)	0.0223 (16)	−0.0130 (15)	0.0158 (15)	−0.0032 (13)
C59	0.0326 (18)	0.0236 (16)	0.0310 (18)	−0.0021 (14)	0.0129 (15)	0.0034 (14)
C60	0.0326 (18)	0.0217 (16)	0.0297 (17)	−0.0009 (13)	0.0169 (15)	0.0009 (13)

### Geometric parameters (Å, °)

**Table:**

Sn1—C1	2.149 (3)	C23—H23	0.9500
Sn1—C15	2.153 (3)	C24—C25	1.473 (3)
Sn1—C8	2.156 (3)	C24—H24	0.9500
Sn1—O1	2.182 (2)	C25—C26	1.3900
Sn1—O2^i^	2.320 (2)	C25—C30	1.3900
Sn2—C45	2.144 (3)	C26—C27	1.3900
Sn2—C38	2.149 (3)	C26—H26	0.9500
Sn2—C31	2.164 (3)	C27—C28	1.3900
Sn2—O5	2.199 (2)	C27—H27	0.9500
Sn2—O6^ii^	2.334 (2)	C28—C29	1.3900
O1—C22	1.267 (3)	C29—C30	1.3900
O2—C22	1.272 (3)	C29—H29	0.9500
O2—Sn1^iii^	2.320 (2)	C30—H30	0.9500
O3—N1	1.222 (6)	C31—C32	1.513 (4)
O4—N1	1.235 (7)	C31—H31A	0.9900
O3'—N1	1.223 (6)	C31—H31B	0.9900
O4'—N1	1.238 (7)	C32—C33	1.3900
O5—C52	1.264 (3)	C32—C37	1.3900
O6—C52	1.259 (3)	C33—C34	1.3900
O6—Sn2^iv^	2.334 (2)	C33—H33	0.9500
O7—N2	1.231 (7)	C34—C35	1.3900
O8—N2	1.240 (7)	C34—H34	0.9500
O7'—N2	1.230 (7)	C35—C36	1.3900
O8'—N2	1.235 (7)	C35—H35	0.9500
N1—C28	1.447 (3)	C36—C37	1.3900
N2—C58	1.446 (3)	C36—H36	0.9500
C1—C2	1.499 (3)	C37—H37	0.9500
C1—H1A	0.9900	C38—C39	1.513 (3)
C1—H1B	0.9900	C38—H38A	0.9900
C2—C3	1.3900	C38—H38B	0.9900
C2—C7	1.3900	C39—C40	1.3900
C3—C4	1.3900	C39—C44	1.3900
C3—H3	0.9500	C40—C41	1.3900
C4—C5	1.3900	C40—H40	0.9500
C4—H4	0.9500	C41—C42	1.3900
C5—C6	1.3900	C41—H41	0.9500
C5—H5	0.9500	C42—C43	1.3900
C6—C7	1.3900	C42—H42	0.9500
C6—H6	0.9500	C43—C44	1.3900
C7—H7	0.9500	C43—H43	0.9500
C8—C9	1.518 (3)	C44—H44	0.9500
C8—H8A	0.9900	C45—C46	1.513 (3)
C8—H8B	0.9900	C45—H45A	0.9900
C9—C10	1.3900	C45—H45B	0.9900
C9—C14	1.3900	C46—C47	1.3900
C10—C11	1.3900	C46—C51	1.3900
C10—H10	0.9500	C47—C48	1.3900
C11—C12	1.3900	C47—H47	0.9500
C11—H11	0.9500	C48—C49	1.3900
C12—C13	1.3900	C48—H48	0.9500
C12—H12	0.9500	C49—C50	1.3900
C13—C14	1.3900	C49—H49	0.9500
C13—H13	0.9500	C50—C51	1.3900
C14—H14	0.9500	C50—H50	0.9500
C15—C16	1.512 (3)	C51—H51	0.9500
C15—H15A	0.9900	C52—C53	1.485 (4)
C15—H15B	0.9900	C53—C54	1.334 (4)
C16—C17	1.3900	C53—H53	0.9500
C16—C21	1.3900	C54—C55	1.468 (3)
C17—C18	1.3900	C54—H54	0.9500
C17—H17	0.9500	C55—C56	1.3900
C18—C19	1.3900	C55—C60	1.3900
C18—H18	0.9500	C56—C57	1.3900
C19—C20	1.3900	C56—H56	0.9500
C19—H19	0.9500	C57—C58	1.3900
C20—C21	1.3900	C57—H57	0.9500
C20—H20	0.9500	C58—C59	1.3900
C21—H21	0.9500	C59—C60	1.3900
C22—C23	1.476 (4)	C59—H59	0.9500
C23—C24	1.327 (4)	C60—H60	0.9500
			
C1—Sn1—C15	116.64 (12)	C26—C25—C30	120.0
C1—Sn1—C8	117.02 (13)	C26—C25—C24	117.50 (17)
C15—Sn1—C8	125.49 (12)	C30—C25—C24	122.48 (17)
C1—Sn1—O1	91.07 (9)	C25—C26—C27	120.0
C15—Sn1—O1	94.51 (10)	C25—C26—H26	120.0
C8—Sn1—O1	93.42 (10)	C27—C26—H26	120.0
C1—Sn1—O2^i^	80.82 (10)	C26—C27—C28	120.0
C15—Sn1—O2^i^	89.00 (10)	C26—C27—H27	120.0
C8—Sn1—O2^i^	90.47 (10)	C28—C27—H27	120.0
O1—Sn1—O2^i^	171.89 (8)	C29—C28—C27	120.0
C45—Sn2—C38	122.28 (12)	C29—C28—N1	119.86 (17)
C45—Sn2—C31	123.99 (12)	C27—C28—N1	120.13 (17)
C38—Sn2—C31	112.99 (12)	C28—C29—C30	120.0
C45—Sn2—O5	94.59 (11)	C28—C29—H29	120.0
C38—Sn2—O5	89.97 (9)	C30—C29—H29	120.0
C31—Sn2—O5	93.75 (10)	C29—C30—C25	120.0
C45—Sn2—O6^ii^	88.74 (10)	C29—C30—H30	120.0
C38—Sn2—O6^ii^	83.09 (10)	C25—C30—H30	120.0
C31—Sn2—O6^ii^	89.43 (10)	C32—C31—Sn2	107.96 (18)
O5—Sn2—O6^ii^	173.04 (8)	C32—C31—H31A	110.1
C22—O1—Sn1	121.17 (19)	Sn2—C31—H31A	110.1
C22—O2—Sn1^iii^	139.0 (2)	C32—C31—H31B	110.1
C52—O5—Sn2	120.44 (19)	Sn2—C31—H31B	110.1
C52—O6—Sn2^iv^	141.3 (2)	H31A—C31—H31B	108.4
O3—N1—O4	126.9 (12)	C33—C32—C37	120.0
O3'—N1—O4'	121.7 (12)	C33—C32—C31	120.21 (19)
O3—N1—C28	118.3 (7)	C37—C32—C31	119.60 (18)
O3'—N1—C28	119.6 (7)	C32—C33—C34	120.0
O4—N1—C28	114.8 (10)	C32—C33—H33	120.0
O4'—N1—C28	118.7 (11)	C34—C33—H33	120.0
O7'—N2—O8'	118 (3)	C33—C34—C35	120.0
O7—N2—O8	130 (2)	C33—C34—H34	120.0
O7'—N2—C58	118.8 (18)	C35—C34—H34	120.0
O7—N2—C58	115.1 (18)	C36—C35—C34	120.0
O8'—N2—C58	122.7 (16)	C36—C35—H35	120.0
O8—N2—C58	114.7 (15)	C34—C35—H35	120.0
C2—C1—Sn1	115.87 (18)	C35—C36—C37	120.0
C2—C1—H1A	108.3	C35—C36—H36	120.0
Sn1—C1—H1A	108.3	C37—C36—H36	120.0
C2—C1—H1B	108.3	C36—C37—C32	120.0
Sn1—C1—H1B	108.3	C36—C37—H37	120.0
H1A—C1—H1B	107.4	C32—C37—H37	120.0
C3—C2—C7	120.0	C39—C38—Sn2	116.21 (18)
C3—C2—C1	120.75 (19)	C39—C38—H38A	108.2
C7—C2—C1	119.23 (19)	Sn2—C38—H38A	108.2
C2—C3—C4	120.0	C39—C38—H38B	108.2
C2—C3—H3	120.0	Sn2—C38—H38B	108.2
C4—C3—H3	120.0	H38A—C38—H38B	107.4
C5—C4—C3	120.0	C40—C39—C44	120.0
C5—C4—H4	120.0	C40—C39—C38	120.74 (17)
C3—C4—H4	120.0	C44—C39—C38	119.26 (17)
C4—C5—C6	120.0	C39—C40—C41	120.0
C4—C5—H5	120.0	C39—C40—H40	120.0
C6—C5—H5	120.0	C41—C40—H40	120.0
C5—C6—C7	120.0	C42—C41—C40	120.0
C5—C6—H6	120.0	C42—C41—H41	120.0
C7—C6—H6	120.0	C40—C41—H41	120.0
C6—C7—C2	120.0	C41—C42—C43	120.0
C6—C7—H7	120.0	C41—C42—H42	120.0
C2—C7—H7	120.0	C43—C42—H42	120.0
C9—C8—Sn1	109.68 (17)	C44—C43—C42	120.0
C9—C8—H8A	109.7	C44—C43—H43	120.0
Sn1—C8—H8A	109.7	C42—C43—H43	120.0
C9—C8—H8B	109.7	C43—C44—C39	120.0
Sn1—C8—H8B	109.7	C43—C44—H44	120.0
H8A—C8—H8B	108.2	C39—C44—H44	120.0
C10—C9—C14	120.0	C46—C45—Sn2	113.29 (19)
C10—C9—C8	120.19 (17)	C46—C45—H45A	108.9
C14—C9—C8	119.81 (17)	Sn2—C45—H45A	108.9
C9—C10—C11	120.0	C46—C45—H45B	108.9
C9—C10—H10	120.0	Sn2—C45—H45B	108.9
C11—C10—H10	120.0	H45A—C45—H45B	107.7
C12—C11—C10	120.0	C47—C46—C51	120.0
C12—C11—H11	120.0	C47—C46—C45	120.02 (19)
C10—C11—H11	120.0	C51—C46—C45	119.97 (19)
C11—C12—C13	120.0	C46—C47—C48	120.0
C11—C12—H12	120.0	C46—C47—H47	120.0
C13—C12—H12	120.0	C48—C47—H47	120.0
C14—C13—C12	120.0	C47—C48—C49	120.0
C14—C13—H13	120.0	C47—C48—H48	120.0
C12—C13—H13	120.0	C49—C48—H48	120.0
C13—C14—C9	120.0	C50—C49—C48	120.0
C13—C14—H14	120.0	C50—C49—H49	120.0
C9—C14—H14	120.0	C48—C49—H49	120.0
C16—C15—Sn1	110.42 (18)	C49—C50—C51	120.0
C16—C15—H15A	109.6	C49—C50—H50	120.0
Sn1—C15—H15A	109.6	C51—C50—H50	120.0
C16—C15—H15B	109.6	C50—C51—C46	120.0
Sn1—C15—H15B	109.6	C50—C51—H51	120.0
H15A—C15—H15B	108.1	C46—C51—H51	120.0
C17—C16—C21	120.0	O6—C52—O5	124.0 (3)
C17—C16—C15	119.98 (18)	O6—C52—C53	119.5 (3)
C21—C16—C15	119.98 (18)	O5—C52—C53	116.4 (3)
C18—C17—C16	120.0	C54—C53—C52	121.9 (3)
C18—C17—H17	120.0	C54—C53—H53	119.0
C16—C17—H17	120.0	C52—C53—H53	119.0
C17—C18—C19	120.0	C53—C54—C55	126.4 (3)
C17—C18—H18	120.0	C53—C54—H54	116.8
C19—C18—H18	120.0	C55—C54—H54	116.8
C20—C19—C18	120.0	C56—C55—C60	120.0
C20—C19—H19	120.0	C56—C55—C54	117.85 (18)
C18—C19—H19	120.0	C60—C55—C54	122.14 (18)
C19—C20—C21	120.0	C57—C56—C55	120.0
C19—C20—H20	120.0	C57—C56—H56	120.0
C21—C20—H20	120.0	C55—C56—H56	120.0
C20—C21—C16	120.0	C56—C57—C58	120.0
C20—C21—H21	120.0	C56—C57—H57	120.0
C16—C21—H21	120.0	C58—C57—H57	120.0
O1—C22—O2	122.5 (3)	C57—C58—C59	120.0
O1—C22—C23	117.2 (3)	C57—C58—N2	119.3 (2)
O2—C22—C23	120.2 (3)	C59—C58—N2	120.7 (2)
C24—C23—C22	121.6 (3)	C60—C59—C58	120.0
C24—C23—H23	119.2	C60—C59—H59	120.0
C22—C23—H23	119.2	C58—C59—H59	120.0
C23—C24—C25	126.7 (3)	C59—C60—C55	120.0
C23—C24—H24	116.6	C59—C60—H60	120.0
C25—C24—H24	116.6	C55—C60—H60	120.0
			
C1—Sn1—O1—C22	175.8 (2)	C28—C29—C30—C25	0.0
C15—Sn1—O1—C22	58.9 (2)	C26—C25—C30—C29	0.0
C8—Sn1—O1—C22	−67.1 (2)	C24—C25—C30—C29	−178.1 (2)
C45—Sn2—O5—C52	62.7 (3)	C45—Sn2—C31—C32	178.15 (17)
C38—Sn2—O5—C52	−174.9 (3)	C38—Sn2—C31—C32	7.8 (2)
C31—Sn2—O5—C52	−61.9 (3)	O5—Sn2—C31—C32	−83.74 (19)
C15—Sn1—C1—C2	94.9 (2)	O6^ii^—Sn2—C31—C32	90.06 (18)
C8—Sn1—C1—C2	−95.0 (2)	Sn2—C31—C32—C33	77.7 (2)
O1—Sn1—C1—C2	−0.6 (2)	Sn2—C31—C32—C37	−97.25 (18)
O2^i^—Sn1—C1—C2	179.2 (2)	C37—C32—C33—C34	0.0
Sn1—C1—C2—C3	−85.2 (2)	C31—C32—C33—C34	−175.0 (2)
Sn1—C1—C2—C7	93.0 (2)	C32—C33—C34—C35	0.0
C7—C2—C3—C4	0.0	C33—C34—C35—C36	0.0
C1—C2—C3—C4	178.2 (2)	C34—C35—C36—C37	0.0
C2—C3—C4—C5	0.0	C35—C36—C37—C32	0.0
C3—C4—C5—C6	0.0	C33—C32—C37—C36	0.0
C4—C5—C6—C7	0.0	C31—C32—C37—C36	175.0 (2)
C5—C6—C7—C2	0.0	C45—Sn2—C38—C39	52.1 (2)
C3—C2—C7—C6	0.0	C31—Sn2—C38—C39	−137.34 (19)
C1—C2—C7—C6	−178.2 (2)	O5—Sn2—C38—C39	−43.3 (2)
C1—Sn1—C8—C9	22.8 (2)	O6^ii^—Sn2—C38—C39	136.2 (2)
C15—Sn1—C8—C9	−168.14 (16)	Sn2—C38—C39—C40	85.3 (2)
O1—Sn1—C8—C9	−70.14 (18)	Sn2—C38—C39—C44	−94.2 (2)
O2^i^—Sn1—C8—C9	102.75 (18)	C44—C39—C40—C41	0.0
Sn1—C8—C9—C10	86.16 (19)	C38—C39—C40—C41	−179.5 (2)
Sn1—C8—C9—C14	−92.93 (18)	C39—C40—C41—C42	0.0
C14—C9—C10—C11	0.0	C40—C41—C42—C43	0.0
C8—C9—C10—C11	−179.1 (2)	C41—C42—C43—C44	0.0
C9—C10—C11—C12	0.0	C42—C43—C44—C39	0.0
C10—C11—C12—C13	0.0	C40—C39—C44—C43	0.0
C11—C12—C13—C14	0.0	C38—C39—C44—C43	179.5 (2)
C12—C13—C14—C9	0.0	C38—Sn2—C45—C46	−5.7 (3)
C10—C9—C14—C13	0.0	C31—Sn2—C45—C46	−175.18 (18)
C8—C9—C14—C13	179.1 (2)	O5—Sn2—C45—C46	87.2 (2)
C1—Sn1—C15—C16	−22.7 (2)	O6^ii^—Sn2—C45—C46	−86.7 (2)
C8—Sn1—C15—C16	168.22 (17)	Sn2—C45—C46—C47	−84.2 (2)
O1—Sn1—C15—C16	70.77 (19)	Sn2—C45—C46—C51	94.3 (2)
O2^i^—Sn1—C15—C16	−101.91 (19)	C51—C46—C47—C48	0.0
Sn1—C15—C16—C17	−83.3 (2)	C45—C46—C47—C48	178.5 (2)
Sn1—C15—C16—C21	94.47 (19)	C46—C47—C48—C49	0.0
C21—C16—C17—C18	0.0	C47—C48—C49—C50	0.0
C15—C16—C17—C18	177.8 (2)	C48—C49—C50—C51	0.0
C16—C17—C18—C19	0.0	C49—C50—C51—C46	0.0
C17—C18—C19—C20	0.0	C47—C46—C51—C50	0.0
C18—C19—C20—C21	0.0	C45—C46—C51—C50	−178.5 (2)
C19—C20—C21—C16	0.0	Sn2^iv^—O6—C52—O5	−176.0 (2)
C17—C16—C21—C20	0.0	Sn2^iv^—O6—C52—C53	3.1 (5)
C15—C16—C21—C20	−177.8 (2)	Sn2—O5—C52—O6	2.2 (4)
Sn1—O1—C22—O2	4.7 (4)	Sn2—O5—C52—C53	−176.9 (2)
Sn1—O1—C22—C23	−175.23 (19)	O6—C52—C53—C54	−167.7 (3)
Sn1^iii^—O2—C22—O1	−175.1 (2)	O5—C52—C53—C54	11.4 (5)
Sn1^iii^—O2—C22—C23	4.8 (5)	C52—C53—C54—C55	176.5 (3)
O1—C22—C23—C24	−2.4 (4)	C53—C54—C55—C56	−169.4 (3)
O2—C22—C23—C24	177.7 (3)	C53—C54—C55—C60	11.8 (5)
C22—C23—C24—C25	177.8 (3)	C60—C55—C56—C57	0.0
C23—C24—C25—C26	−179.2 (3)	C54—C55—C56—C57	−178.8 (2)
C23—C24—C25—C30	−1.0 (4)	C55—C56—C57—C58	0.0
C30—C25—C26—C27	0.0	C56—C57—C58—C59	0.0
C24—C25—C26—C27	178.2 (2)	C56—C57—C58—N2	178.0 (2)
C25—C26—C27—C28	0.0	O7'—N2—C58—C57	177.1 (10)
C26—C27—C28—C29	0.0	O7—N2—C58—C57	−167.7 (10)
C26—C27—C28—N1	179.1 (2)	O8'—N2—C58—C57	−3.0 (10)
O3—N1—C28—C29	−18.7 (4)	O8—N2—C58—C57	12.3 (10)
O3'—N1—C28—C29	8.5 (4)	O7'—N2—C58—C59	−4.9 (10)
O4—N1—C28—C29	160.8 (5)	O7—N2—C58—C59	10.2 (10)
O4'—N1—C28—C29	−171.4 (4)	O8'—N2—C58—C59	175.0 (10)
O3—N1—C28—C27	162.2 (4)	O8—N2—C58—C59	−169.8 (10)
O3'—N1—C28—C27	−170.6 (4)	C57—C58—C59—C60	0.0
O4—N1—C28—C27	−18.3 (5)	N2—C58—C59—C60	−177.9 (2)
O4'—N1—C28—C27	9.5 (4)	C58—C59—C60—C55	0.0
C27—C28—C29—C30	0.0	C56—C55—C60—C59	0.0
N1—C28—C29—C30	−179.1 (2)	C54—C55—C60—C59	178.7 (3)

Symmetry codes: (i) −*x*+3/2, *y*+1/2, −*z*+3/2; (ii) −*x*+1/2, *y*+1/2, −*z*+3/2; (iii) −*x*+3/2, *y*−1/2, −*z*+3/2; (iv) −*x*+1/2, *y*−1/2, −*z*+3/2.
